# Did diet compliance and remission reduce oxidative stress in celiac patients?

**DOI:** 10.1590/1806-9282.20231120

**Published:** 2024-05-03

**Authors:** Berat Ebik, Ferhat Bacaksiz, Ali Uzel, Mustafa Zanyar Akkuzu, Ahmet Yavuz, Huseyin Kacmaz, Nihat Aslan, Medeni Arpa, Salim Neselioglu, Ozcan Erel

**Affiliations:** 1 University of Health Sciences, Diyarbakır Gazi Yasargil Education and Research Hospital, Division of Gastroenterology – Diyarbakır, Turkey.; 2 Adıyaman University, Faculty of Medicine, Department of Gastroenterology – Adıyaman, Turkey.; 3 Ministry of Health, Batman Training and Research Hospital, Internal Medicine Clinic – Diyarbakır, Turkey.; 4 Recep Tayyip Erdoğan University, School of Medicine, Department of Biochemistry – Rize, Turkey.; 5 Ankara Yıldırım Beyazıt University, Faculty of Medicine, Department of Biochemistry – Ankara, Turkey.

**Keywords:** Celiac disease, Oxidative stress, Thiol-disulfide

## Abstract

**OBJECTIVE:**

We aimed to examine the effect of remission status on thiol–disulfide homeostasis in celiac patients and thus to indirectly determine the effect of oxidative stress and inflammation caused by non-compliance with the diet.

**METHODS:**

Between February 2019 and December 2021, 117 patients diagnosed with celiac disease were included in this prospective randomized and controlled study. In addition to routine tests of celiac patients, thiol and disulfide measurements were made from the blood both at the beginning of the study and at the end of the first year.

**RESULTS:**

While 52 of the patients (44.4%) were in remission, 65 patients (55.6%) were not. There was an evident increase in native thiol levels of the patients who were initially not in remission but went into at the end of the first year (347.4±46.7 μmol/L vs. 365.3±44.0 μmol/L; p=0.001). Mean plasma disulfide levels of patients with celiac going into remission became reduced in the first year from the level of 14.5±5.1 μmol/L down to 8.9±4.2 μmol/L (p<0.001). In celiac patients who entered remission, disulfide and anti-tissue transglutaminase immunoglobulin A levels decreased in a correlation (r=0.526; p<0.001).

**CONCLUSION:**

Not being in remission in celiac disease leads to increased oxidative stress, and thiol–disulfide homeostasis is an indirect indicator of this. Additionally, providing remission in celiac patients reduces oxidative stress.

## INTRODUCTION

Celiac disease is an immune-mediated systemic disease triggered by the ingestion of gluten and related prolamins found in wheat, barley, rye, and oats in genetically susceptible individuals. People with HLA-DQ2 and HLA-DQ8 genotypes develop autoantibodies against tissue transglutaminase (tTG) enzyme, which plays an important role in pathogenesis, after gluten intake^1^. The prevalence of celiac disease worldwide is estimated to be 1%^2^.

In CD patients, there is a proinflammatory response accompanied by intraepithelial lymphocytosis, crypt hyperplasia, and villus atrophy, caused by the activation of antigen-specific T lymphocytes induced by HLADQ2 and DQ8. As celiac patients are exposed to gluten, the production of interleukin (IL)-15, IL-18, and IL-21 increases. This causes an ongoing inflammatory response^3^.

Thiol–disulfide hemostasis (TDH) is one of the antioxidant systems of the body. When oxidative damage occurs due to reactive oxygen species (ROS), sulfide-containing thiol groups are oxidized, and disulfide bonds are formed. Disulfide bonds are dynamic covalent bonds that are formed between two thiol groups. These are two-way reactions and the thiol groups formed are re-reduced to thiol groups by redox reaction, and dynamic TDH is maintained^4,5^.

In this study, we investigated whether there is a difference in terms of TDH between celiac patients who are in remission and those who are not. We also examined the changes in thiol and disulfide levels in both remitted and non-remitted celiac patients. Thus, we aimed to indirectly evaluate the effect of chronic inflammation caused by noncompliance with the diet on oxidant and antioxidant balance and ROS-related oxidative stress through TDH.

## METHODS

### Study design and participants

Our study was designed as a prospective randomized controlled study. A total of 117 patients diagnosed with celiac disease presenting to the gastroenterology polyclinic between February 2019 and December 2021 were included in our study.

### Ethical considerations

The patients participating in the study were informed about the aim of the study. Written informed consent was requested from the patients to participate in the study. Patients who signed the informed consent form were included in the study. This study was approved by the decision of the University of Health Sciences, Diyarbakır Gazi Yaşargil Training and Research Hospital Clinical Research Ethics Committee dated 26.03.2021 and numbered 719.

### Inclusion criteria

Having a diagnosis of celiac disease (patients who were previously positive for celiac autoantibodies (anti-transglutaminase immunoglobulin A (IgA) or anti-endomysium IgA) and were diagnosed with celiac disease according to the MARSH classification by endoscopic biopsy^6^), being over 16 years of age, patients who agree to give a blood sample for thiol–disulfide level in addition to routine laboratory tests at the beginning and end of the study, having normal WBC and CRP levels, and normal albumin level (as it may affect the thiol–disulfide level^7^).

### Exclusion criteria

Celiac patients younger than 16 years of age, those with chronic systemic diseases (such as hypertension, diabetes mellitus, heart failure, chronic kidney disease, chronic lung disease, and chronic liver disease), patients with active infection, those with a disease that may cause low albumin in the blood^5^, smoking or alcohol^6^ users, pregnant celiac patients, patients using antioxidant vitamins and/or herbal products, and patients who did not sign the informed consent form were excluded from the study.

### Thiol–disulfide measurement

In our study, native thiol and total thiol concentrations were measured synchronously as a paired test. In the first container, the amount of native thiol groups was measured by using a modified Ellman reagent. In a parallel study, first of all, dynamic disulfide bonds were reduced to free thiol groups by sodium borohydride. It was removed by NaBH4 formaldehyde in order to prevent the unused reduced sodium borohydride from reduction into 5,5´-dithio-bis-2-nitrobenzoic acid (DTNB). After reaction with DTNB, native thiol (NT) and total thiol (TT) levels were determined, and eventually the levels were measured. The result obtained by subtracting the amount of native thiol from the total thiol content and thereafter dividing it by half indicated the disulfide (DS) level^8^.

Reference range determined for native thiol (-SH) is 278–826 μmol/L, for total thiol ((S-S)+(-SH)), it is 441–740 μmol/L, and for disulfide (S-S), it is 2–52 μmol/L^9^.

When disulfide, native thiol, and total thiol levels were divided by each other, disulfide/native thiol, disulfide/total thiol, and native thiol/total thiol rates were obtained. Disulfide/native thiol (sD/sNT), disulfide/total thiol (sD/sTT), and native thiol/total thiol (sNT/sTT) rates were calculated in percentages (%).

### Other laboratory tests

When celiac patients come for control, as recommended by the guidelines^6^, complete blood count, ferritin, iron, iron-binding capacity, folic acid, vitamin B12, vitamin D, calcium, phosphorus, magnesium, albumin, glucose, urea, creatinine, sodium, potassium, liver function tests, parathormone and DEXA for measuring bone mineralization, thyroid hormones and autoantibodies, and celiac autoantibody levels were checked.

### Evaluation of remission

Patients who state that they are on a gluten-free diet (for at least 6 months) were asymptomatic, and those who had the tTG IgA level below the cutoff value of 12 U/mL were considered to be in clinical remission and asymptomatic celiac disease. Patients who were noncompliant with the diet, had malabsorption symptoms, had anemia, vitamin, and mineral deficiency in laboratory parameters, and had tTG IgA level>12 U/mL were clinically evaluated as symptomatic CD. Due to its high specificity, IgA-EMA was used as a confirmation test, especially in cases where tTG IgA had a low titer. Autoantibodies were used to assess compliance with the gluten-free diet^10^.

Laboratory parameters of remission and non-remission patients at the baseline and native and total thiol levels as well as disulfide level and rates thereof were statistically compared. Patients who were non-remission at the baseline and patients coming for control a year later were classified based on their state of remission. Native and total thiol levels among these groups as well as disulfide levels and rates thereof were compared with the values of these patients measured at the baseline.

The aim of using this method was to uncover the effect of dietary adherence and remission on thiol–disulfide hemostasis in patients with CD.

### Statistical analysis

Kolmogorov-Smirnov, Shapiro-Wilk test, coefficient of variation, and skewness and kurtosis methods were used to control the normal distribution of patient data. While mean and standard deviation values were stated for continuous variables, categorical variables were expressed as %. Independent t-test or Mann-Whitney U test was used to determine the difference between age, body mass index, dietary adherence, and laboratory parameters between remission and non-remission patients with celiac disease. Paired samples t-test was used for parameters that had normal distribution, and Wilcoxon test was used for parameters that did not have normal distribution to determine the differences between the native and total thiol and disulfide levels and their ratios to each other at the baseline and 1 year later in non-remission celiac patients. Pearson correlation analysis was performed to show the relationship between disulfide and anti-Ttg IgA levels at baseline and in the first year of remission patients with celiac disease. All tests were bilateral, and p<0.05 was considered statistically significant. Statistical analyses were performed by using the package program SPSS24.0 for Windows (SPSS Inc., Chicago, IL, USA).

## RESULTS

Demographic and laboratory data of celiac patients in remission and non-remission are shown in Table 1. While 52 (44.4%) of 117 patients with celiac included in the study were in remission, 65 patients (55.6%) were not. The mean age of the patients was 30.7±10.8. Of the patients, 60 (51.3%) were female and 57 (48.7%) were male. Although 71.8% (n=84) of the patients stated that they were on a diet, 38.1% (n=32) of these patients were not in remission. The mean anti-Ttg IgA value was 7.5±5.8 U/mL in remission patients with CD, but the value was 205.3±124.3 U/mL in non-remission patients (p<0.001).

**Table 1:** Demographic and laboratory data of celiac patients in remission and non-remission.

	In remission (n=52)	Not in remission (n=65)	p
Age (years)	35.1±13.7	27.3±8.5	0.001
Gender F/M	24 (46.2%)/28 (53.8%)	36 (55.4%)/29 (44.6%)	0.433
Diet compliance Yes/No	52 (100%)	32 (49.3%)/33 (50.7%)	<0.001
Anti Ttg Ig A (U/mL)	7.5±5.8	205.3±124.3	<0.001
BMI (kg/m^2^)	22.2±1.8	20.1±3.8	<0.001
Albumin (g/L)	4.5±0.4	4.4±0.3	0.193
Hgb (g/dL)	14.7±1.9	13.3±2.4	0.005
Iron (μg/dL)	21.4±18.6	8.1±6.1	<0.001
Ferritin (μg/L)	60.6±40.9	20.3±16.5	<0.001
Folate (ng/mL)	10.7±10.2	6.2±4.3	0.026
Vitamin D (ng/mL)	19.1±9.2	18.7±8.4	0.745
Calcium (mg/dL)	9.6±0.6	9.0±0.4	0.294
Phosphorus (mg/dL)	3.4±0.7	3.2±0.5	0.149
DEXA (Z score)	-0.5±1.2	-1.5±1.2	<0.001
WBC (10^3^ cell/•1)	7100±1500	7400±1100	0.315
CRP (mg/L)	0.6±0.2	0.5±0.3	0.254

BMI: body mass index; Anti Ttg IgA: anti-transglutaminase antibodies A; Hgb: hemoglobin; DEXA: dual-energy X-ray absorptiometry; WBC: white blood cell; CRP: C-reactive protein.

The mean plasma total thiol level was 374.4±46.5 μmol/L at the baseline in celiac patients in remission, while it was 392.7±43.6 μmol/L at the end of the first year (p<0.001). In patients who did not go into remission, mean plasma total thiol levels were similar at the baseline and at the first year (382.1±34.5 μmol/L vs. 383.1±36.0 μmol/L; p=0.392). While the mean plasma disulfide level of celiac patients in remission decreased from 14.5±5.1 μmol/L to 8.9±4.2 μmol/L in the first year (p<0.001), no change was observed in celiac patients who did not go into remission (17.6±3.1 μmol/L vs. 17.7±3.2; p=0.784). In addition, serum disulfide/native thiol and disulfide/total thiol ratios reached lower levels in celiac patients who went into remission (Table 2).

**Table 2:** The difference between the thiol–disulfide values 1 year later in celiac patients who were not in remission at the beginning but achieved remission after adhering to the recommended diet, and those who did not comply with the diet and could not achieve remission.

	In remission (n=38)			Not in remission (n=27)		
	Initially	1 year	p	Initially	1 year	p
Native thiol (μmol/L)	347.4±46.7	365.3±44.0	0.001	352.9±30.2	353.5±30.6	0.654
Total thiol (μmol/L)	374.4±46.5	392.7±43.6	<0.001	382.1±34.5	383.1±36.0	0.392
Disulfide (μmol/L)	14.5±5.1	8.9±4.2	<0.001	17.6±3.1	17.7±3.2	0.784
(Disulfide/native thiol)x100 (%)	3.9±1.6	3.6±1.5	0.022	5.5±0.5	5.6±0.6	0.688
(Disulfide/total thiol)x100 (%)	3.8±1.4	3.3±1.4	0.015	4.5±0.4	4.4±0.5	0.559
(Native thiol/total thiol)x100 (%)	92.7±1.8	93.0±2.1	0.772	92.0±1.9	92.2±1.7	0.450

**Figure 1:**
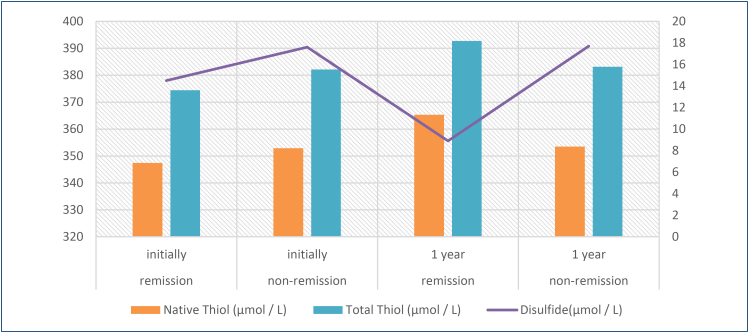
Cumulative change of thiol and disulfide levels in celiac patients in remission compared to non-remission patients.

Among the celiac patients who were not in remission at the baseline, there were significant decreases in both plasma disulfide and anti-transglutaminase IgA levels at the end of the first year in patients who adhered to the diet and went into remission (p<0.001). Furthermore, disulfide and anti-tTG IgA levels decreased in a correlational manner while the patients were in remission (r=0.526; p<0.001). It was observed that in patients with similar total thiol levels at the beginning, a significant increase occurred in patients who were on diet compared with patients who were not on diet, due to the effect of long-term remission (Figure 1).

## DISCUSSION

Extracellular redox reactions can regulate tissue homeostasis via its effects on cell proliferation, differentiation, apoptosis, and immune system. Therefore extracellular redox and thiol/disulfide balance, an important component thereof, has a significant impact on, in particular, diseases with an inflammatory progression^11^.

In our study, we did not notice any differences with regard to total and native thiol levels between remission and non-remission patients with celiac disease. The underlying reason is that thiol level is affected by many factors including body mass index and that total thiol capacity of each individual is different^12^. However there were significant differences between both groups in terms of disulfide level. Moreover, thiol:disulfide ratios were different. This case may be rather an indicator of an ongoing inflammation constantly triggered by gluten exposure in non-remission celiac patients than total thiol content.

The most important result we reached in this study is the increase in the body's total thiol pool as well as the decrease in disulfide levels in compliance with the gluten-free diet in patients who were initially non-compliant with the diet and were not in clinical remission. This result shows that the gluten-free diet reduces inflammation in the body and increases antioxidant capacity. It was not possible to say the same for patients who were non-compliant with the diet. Even after a year, no significant changes were observed in total thiol and disulfide levels in the body.

A study conducted thereon states that in these patients thiol absorption is not impaired, and TDH is dependent on gliadin toxicity and auto-inflammation^13^. We have the same opinion in regard to this matter. Should thiol absorption be impaired, there would be significant differences between remission and non-remission celiac patients. There could also be a difference between both groups with regard to albumin levels.

However, the fact that the total thiol pool is different for everyone and that these biomarkers have not been compared with methods that directly measure ROS prevents us from reaching a clear conclusion on this subject. A meta-analysis comparing the tests related to oxidative stress may provide us with further opinions.

Nevertheless, we can say that disulfide level and anti-transglutaminase antibody decreased in a correlated manner in patients going into remission at the end of the first year. Anti-transglutaminase antibodies are essentially produced against the inflammation due to gliadin exposure, and against the tissue transglutaminase at the end of deamidation reactions, and in parallel, a large amount of cytokines are released from CD4 T lymphocytes^14,15^. In fact, anti-transglutaminase and disulfide levels reduced with the diet may be an indirect indicator of a reduction in cytokine levels.

### Study limitations

The most important limitation of our study was that we could not measure the levels of cytokines such as interleukin and interferon, which are inflammatory parameters. Unfortunately, there are no any such studies in literature either. If we were able to measure these parameters and had a chance to compare them with thiol and sulfide levels, we could obtain more and detailed data about ROS with both the chronic inflammatory situation in CD and the oxidative stress caused by remission. In addition, since the majority of our patients were reluctant to undergo endoscopy, histopathological examination of the duodenal mucosa, which is recommended as the gold standard^16^ in determining the remission status of patients, could not be performed.

## CONCLUSION

In CD, not being in remission, especially non-compliance with the diet, causes increased oxidative stress. TDH is an indirect indicator of this. Additionally, achieving remission in celiac patients by complying with a gluten-free diet reduces oxidative stress in the body. Maintaining remission in CD by complying with a gluten-free diet may be protective against the risk of developing other chronic and autoimmune diseases.
